# Fabrication of Dual-Phase Mixed Conductor Four-Channel Hollow Fiber Membrane for Hydrogen Separation

**DOI:** 10.3390/membranes16050158

**Published:** 2026-04-30

**Authors:** Doudou Jia, Haonan Wang, Zhengkun Liu, Guangru Zhang, Wanqin Jin

**Affiliations:** 1 State Key Laboratory of Materials-Oriented Chemical Engineering, College of Chemical Engineering, Nanjing Tech University, Nanjing 211816, China; jiadoudou@njtech.edu.cn (D.J.); wanghaonan@njtech.edu.cn (H.W.); guangru.zhang@njtech.edu.cn (G.Z.); wqjin@njtech.edu.cn (W.J.); 2 Quzhou Membrane Material Innovation Institute, 99 Zheda Rd, Quzhou 324000, China

**Keywords:** multi-channel hollow fiber, dual-phase membrane, hydrogen separation, mixed conductor

## Abstract

Perovskite mixed proton–electron hydrogen-permeable membranes have been widely applied in the field of membrane separation due to their 100% selectivity for hydrogen separation. La_5.5_WO_11.25-δ_-La_0.87_Sr_0.13_CrO_3-δ_ (LWO-LSF) four-channel hollow fiber membranes were prepared by the phase inversion and sintering technique using a one-step thermal processing (OSTP) approach. The effects of temperature, feed gas concentration, sweep gas flow, permeation mode, and water vapor on hydrogen flux were systematically investigated. At 900 °C, the hydrogen permeation flux of 50% H_2_/N_2_ feed from the shell side to the lumen side was 0.613 mL·min^−1^·cm^−2^, which was 62.59% higher than that from the lumen side to the shell side. The enhanced hydrogen permeation performance is attributed to the lower gas mass transfer resistance under shell-side feeding. Under humidified conditions on the sweep side, the hydrogen flux increased by an additional 3.42%. The presence of water vapor increased the number of proton carriers, effectively enhancing proton–electron-coupled transport and thereby increasing the hydrogen permeation flux.

## 1. Introduction

Hydrogen, as a clean, carbon-free, renewable fuel, can be used as a substitute for traditional fossil fuels in addressing global climate change [1,2,3]. At present, the main production methods of hydrogen are steam reforming of natural gas, coal gasification, and the water–gas shift reaction [4,5,6]. However, the gray hydrogen produced by this method cannot be used directly, as it typically contains a range of impurities, such as CO, CO_2_, H_2_S, and CH_4_ [7,8]. Therefore, the separation and purification of hydrogen become particularly important.

Traditional hydrogen separation and purification technologies primarily include pressure swing adsorption (PSA) [9,10,11,12,13] and cryogenic separation [14,15,16]. Among these, PSA relies on the distinct adsorption affinities of solid adsorbents for different gas components, as well as on the pressure-dependent variation in the adsorption capacity of each component. Hydrogen separation via PSA technology can produce ultrapure hydrogen with a purity exceeding 99.999%. Nevertheless, this process suffers from a critical drawback: pressure release during the desorption stage results in considerable hydrogen loss (approximately 20%), yielding a hydrogen recovery rate of only 80% [17]. The cryogenic distillation method achieves gas fractionation by exploiting the substantial differences in boiling points among the components of the feed gas. In practical industrial operation, the cryogenic separation process requires the configuration of gas compressors and cryogenic cooling facilities. These pieces of equipment are not only bulky but are also highly energy-intensive, leading to relatively high overall operational and maintenance costs for the process [18]. However, membrane separation technology, with low energy consumption and no phase change, has received widespread attention.

Metal membranes represented by Pd and Pd alloy (23% Ag) membranes can prepare ultra-high purity hydrogen. However, hydrogen embrittlement and a significant reduction in hydrogen permeability in gases containing H_2_S and CO have hindered their development [19,20]. It is widely acknowledged that such chemical instability can be effectively mitigated through material optimization or structural design (such as hollow fiber configuration) [21]. Dense mixed proton–electron conducting ceramic membranes represent an economical and efficient purification process. They can selectively permeate H_2_ at high temperatures and possess excellent high-temperature stability and chemical tolerance. They have great potential for H_2_ purification and separation.

ABO_3_-type perovskite oxides constitute a widely studied class of mixed proton–electron conducting ceramics, with a theoretical hydrogen selectivity of 100%. Among them, doped SrCeO_3_, BaCeO_3_, SrZrO_3_, and BaZrO_3_ systems have attracted extensive research attention [22,23,24,25]. Considerable efforts have been made by researchers to enhance hydrogen permeation flux, mainly through developing novel membrane materials and structural configurations. Escolastico et al. reported that the H_2_ flux of 0.095 mL·min^−1^·cm^−2^ was achieved through the La_5.5_W_0.8_Re_0.2_O_11.25-δ_ membrane at 700 °C under 100 mL·min^−1^ of 50% H_2_/He and humidified sweep gas conditions (all configurations pH_2_O = 0.025 atm) [26]. Unfortunately, the H_2_ flux is uncompetitive and insufficient for their industrial applications. The dual-phase membrane can provide sufficient protons and electrons and, compared with the single-phase hydrogen-permeable membrane, exhibits a higher hydrogen permeation rate. The metal–ceramic dual-phase membrane exhibits poor thermal compatibility and chemical stability, and its long-term stability results are unsatisfactory. However, the ceramic–ceramic dual-phase membrane has been widely studied. For example, Escolástico S et al. have prepared dual-phase La_5.5_WO_11.25-δ_-La_0.87_Sr_0.13_CrO_3-δ_ (LWO-LSC) membranes by solid-phase ball milling with different ratios. A H_2_ flux of 0.15 mL·min^−1^·cm^−2^ was obtained using a 370 μm disk membrane at 700 °C, with 50 vol% H_2_-He (100 mL·min^−1^) as the feed gas and humidified Ar (150 mL·min^−1^, pH_2_O = 0.025 atm) as the sweep gas under the H_2_/humidified Ar gradient [27]. REBOLLO E et al. have systematically studied a novel dual-phase BaCe_0.65_Zr_0.20_Y_0.15_O_3-δ_-Ce_0.85_M_0.15_O_2-δ_ hydrogen-permeable membrane and found a notably stable H_2_ flux of 0.27 mL·min^−1^·cm^−2^ was achieved at 755 °C under a H_2_/humidified Ar partial pressure gradient, with 50 vol% H_2_-He (100 mL·min^−1^) as the feed gas and humidified Ar (150 mL·min^−1^, pH_2_O = 0.03 atm) as the sweep gas [28]. Cheng et al. have prepared a BaCe_0.5_Fe_0.5_O_3-δ_ precursor that decomposed automatically into a dual-phase material, which showed an extremely high hydrogen permeation flux through the 1.0 mm-thick BCF8515-BCF1585 dual-phase membrane reached 0.76 mL·min^−1^·cm^−2^ at 950 °C, with 50 vol% H_2_-50 vol% He (100 mL·min^−1^) as the feed gas and dry Ar (60 mL·min^−1^) as the sweep gas under dry atmosphere [29].

While the introduction of a secondary phase greatly improves hydrogen flux in dual-phase ceramic membranes, disk membranes limit their performance and hinder industrial application [30]. Wang et al. prepared seven-channel hollow fiber membranes via a one-step thermal treatment, which simultaneously enhanced hydrogen permeation flux and mechanical strength [31]. Nevertheless, the seven-channel architecture involves complicated fabrication processes. To balance hydrogen permeation flux, fabrication feasibility, and mechanical strength, this work combines the merits of dual-phase disk membranes and single-phase multi-channel membranes. A dual-phase, four-channel hollow fiber membrane with excellent hydrogen permeation performance and structural stability is fabricated via a phase-inversion, one-step thermal treatment process.

Therefore, in this work, lanthanum tungstate La_5.5_WO_11.25-δ_ (LWO) and lanthanum strontium ferrite La_0.87_Sr_0.13_FeO_3-δ_ (LSF) were used as the proton-conducting phase and oxygen-ion–electron-conducting phase, respectively. A La_5.5_WO_11.25-δ_-La_0.87_Sr_0.13_CrO_3-δ_ (LWO-LSF) dual-phase four-channel hollow fiber membrane was fabricated via phase inversion–one-step thermal treatment. The influences of temperature, feed gas concentration, hydrogen permeation mode, and water vapor on permeation flux were studied. The membrane exhibits stable hydrogen permeation flux in H_2_-containing reducing and humid atmospheres, showing promising application prospects.

## 2. Materials and Methods

### 2.1. LWO-LSF Hollow Fiber Fabrication

The 50 wt–50 wt% LWO-LSF four-channel hollow fiber membranes were prepared by the phase inversion and sintering technique using a one-step thermal processing (OSTP) approach [32]. Fe_2_O_3_ (McClain, Shanghai, China, 99.9%), SrCO_3_ (Sinopharm Chemical Reagent Co., Ltd., Shanghai, China, 99.0%), La_2_O_3_ (Aladdin, Shanghai, China, 99.99%), and WO_3_ (Sinopharm Chemical Reagent Co., Ltd., Shanghai, China, 99.0%) were weighed according to the stoichiometric ratios of La_5.5_WO_11.25-δ_ and La_0.87_Sr_0.13_FeO_3-δ_ to prepare LWO and LSF raw material powders, respectively. Then, based on the 50 wt–50 wt% LWO-LSF ratio calculation, LWO and LSF raw material powders were weighed at a mass ratio of 51.05:50.10. The powder mixture was then wet-milled in ethanol for 24 h at a rotational speed of 300 rpm, and subsequently dried in an oven at 85 °C for 24 h. The mixture was ground and sieved through a 300-mesh sieve to obtain the precursor powder. The precursor powder was mixed with the organic solvent 1-methyl-2-pyrrolidone (AR, Aladdin) and the polymer binder polyetherimide (99.5%, Aladdin) in a mass ratio of 7:4:1, with 1.0 wt% polyvinylpyrrolidone (Sinopharm Chemical Reagent Co., Ltd., Shanghai, China, 99.0%) added to increase fluidity. The spinning solution was then ball-milled at 285 rpm for 24 h using a four-channel spinneret. The iron-red four-channel hollow fiber membrane billet was obtained and then air-dried at room temperature for 24 h. The details of the fabrication procedures have been described elsewhere [33]. Green membranes were placed in an air atmosphere furnace and sintered at 1580 °C for 10 h to obtain a dense four-channel hollow fiber membrane.

To provide contrast, 50 wt–50 wt% LWO-LSF powder was prepared by isostatic pressing to fabricate LWO-LSF disk membranes. A total of 1.2 g of LWO-LSF dual-phase powder was pressure-pressed at 10 MPa for 1 min. Dense disk-shaped membranes were sintered at 1580 °C for 10 h in the high-temperature furnace.

### 2.2. Characterization

X-ray diffraction (XRD, Rigaku, Miniflex 600, Akishima, Japan) with Cu Kα radiation (*λ* = 0.15406 nm) was used to characterize the phase structures of the powders and membranes. At room temperature, the scanning range was from 20° to 80°, with an increment of 0.02°. The morphologies of disk-shaped membranes and hollow fiber membranes were characterized by scanning electron microscopy (SEM, Hitachi S-4800, Tokyo, Japan). The elemental composition of the membrane materials was determined by energy dispersive X-ray spectroscopy (EDX, Hitachi S-4800, Tokyo, Japan) in conjunction with the electron microscope. In a dynamic air atmosphere ranging from room temperature to 1500 °C, the sintering behavior of the LWO-LSF dual-phase membrane was investigated using a thermal expansion instrument (Netzsch DIL 402 C, Hanau, Germany) at a heating rate of 5 °C·min^−1^.

### 2.3. Hydrogen Permeation Test

**Figure 1:**
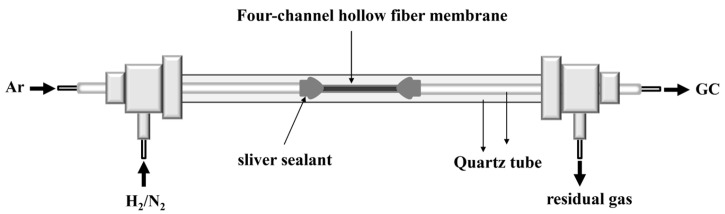
Schematic diagram of the H_2_ permeation apparatus for LWO-LSF hollow fiber membrane in the SL mode.

The hydrogen permeation performance of the dense LWO-LSF four-channel hollow fiber membrane was measured in a home-made high-temperature setup (Figure 1), as described in our previous work [34]. Firstly, the hollow fiber membrane with a length of 30 mm was sealed between two quartz tubes (inner diameter = 2.5 mm, outer diameter = 3.8 mm) using silver glue (Shanghai Synthetic Resin Institute, DAD-84, Shanghai, China). To ensure a secure connection, it was dried in an oven at 120 °C for 2 h. The effective permeation length was 15 mm after deducting the length of the sealed part. Then it was placed inside another quartz tube (diameter = 8 mm, outer diameter = 12 mm) to form the shell side.

For H_2_ permeation measurement, the H_2_/N_2_ gas mixture was fed into the hollow fiber lumen side, and Ar as the sweep gas was fed into the shell side—the operating temperature ranged from 700 to 900 °C. The flow rates of the H_2_/N_2_ feed gas and the Ar sweep gas were controlled by mass flow controllers (Model CS200, Sevenstar, China). The Ar on the sweep side carried the permeated H_2_ into the gas chromatograph (GC9860-5C-NJ, Nanjing Haorupu, China, equipped with a Porapak N column and a TCD detector, using Ar as the carrier gas) for analyzing the gas composition.

H_2_ flux (J_H2_) is calculated according to the following formula [35]. Slight gas leakage may occur at the setup seals, yet the nitrogen leakage rate remained below 5% throughout permeation tests. The leakage fraction has been corrected in hydrogen flux calculations, guaranteeing the reliability of the results. In Equation (1), Cs(N_2_) and Cs(H_2_) represent the concentrations of N_2_ and H_2_ on the sweep side, F_N2_, F_H2_, and F represent the outlet concentrations of N_2_, H_2_, and the total flow rate, respectively. Due to the complex structure of hollow fiber membranes, A is defined herein as the outer surface area of the membrane.

(1) $$J_{H_{2}}=\left(C_{S}\left(H_{2}\right)\;-\;\frac{{\sqrt{14}F_{H_{2}}\;\times \;C}_{S}\left(N_{2}\right)}{F_{N_{2}}}\right)\;\times \;\frac{F}{A}$$

## 3. Results

### 3.1. Phase Structure

**Figure 2:**
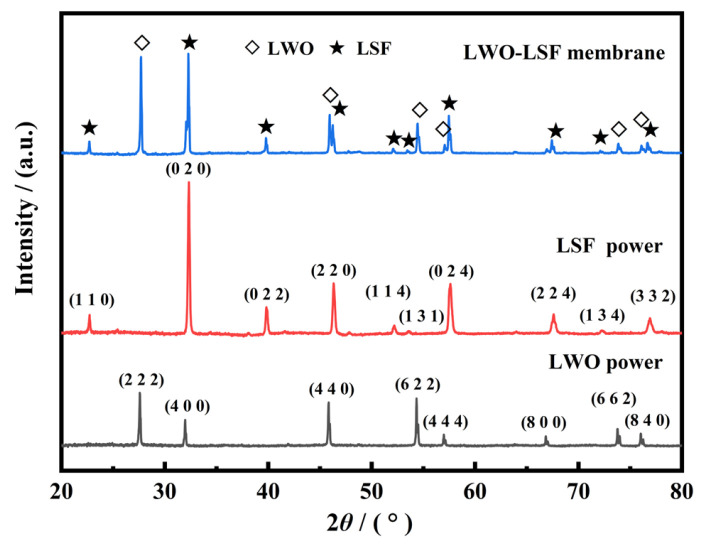
XRD patterns of LWO and LSF powders and LWO-LSF dual-phase hollow fiber membrane sintered at 1580 °C for 10 h.

After calcination, the crystal structure of the LWO-LSF hollow fiber membrane was determined by XRD. Figure 2 shows the XRD pattern of the LWO-LSF dual-phase membrane after sintering at 1580 °C for 10 h in a static air atmosphere. A dual-phase structure consisted of a LWO phase (PDF#16-0391) with fluorite structure [36] and an LSF phase (PDF#35-1480) with perovskite structure [37]. No impurity peaks or second phases were observed in the XRD, indicating good chemical compatibility between the LWO and LSF phases. Moreover, the diffraction peaks in the spectrum were sharp, suggesting that the LWO, LSF powders, and LWO-LSF dual-phase membrane all have high crystallinity, which usually contributes to the improvement of proton lattice conduction.

### 3.2. Morphologies

**Figure 3:**
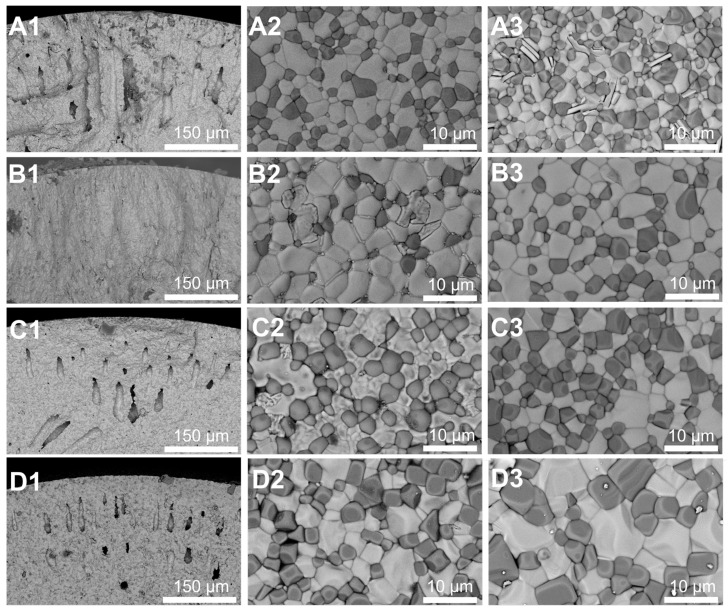
SEM images of the sintered LWO-LSF hollow fiber membranes with different temperatures. (**A1**–**A3**) 1500 °C; (**B1**–**B3**) 1530 °C; (**C1**–**C3**) 1550 °C; (**D1**–**D3**) 1580 °C; (**A1**–**C1**) cross-section; (**A2**–**C2**) outside surface; (**A3**–**C3**) inside surface.

Figure 3 presents the cross-sectional structures (A1–D1) and outside surface (A2–D2) and inside surface (A3–D3) SEM images of LWO-LSF dual-phase hollow fiber membranes sintered at 1500–1580 °C. At 1500 °C, the sintering process remained relatively incomplete, with finger-like pores still well preserved. As the temperature was increased to 1580 °C, grain boundary migration accelerated, and particle densification was promoted, leading to the disappearance of the porous structure within the sponge-like region and the formation of a dense microstructure. Meanwhile, the finger-like pores shrank in size yet maintained their structural integrity. Owing to the radial sintering gradient across the membrane wall, the inner and outer surfaces of the hollow fiber membrane exhibit an asymmetric sintering morphology, which is pronounced at low sintering temperatures and diminishes at higher temperatures. The outer surface, directly exposed to the furnace atmosphere, shows faster sintering kinetics and grain growth, while thermal hysteresis delays densification on the inner surface. This microstructural gradient has no negative effect on the membrane’s bulk homogeneity or hydrogen permeation performance. From the outer surface (A2–D2) and the inner surface (A3–D3) of the membrane, we observe that the size of the two-phase grains has increased, with increasingly distinct grain boundaries. The outer and inner surfaces show consistent evolution, confirming uniform sintering across the multi-channel structure.

**Figure 4:**
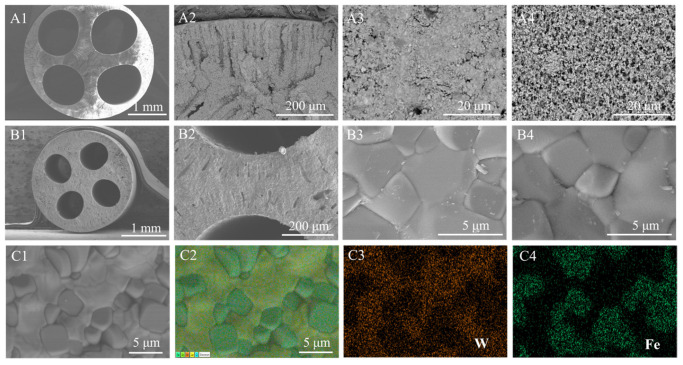
Cross-sections of the LWO-LSF hollow fiber membrane: (**A1**,**B1**); magnified cross-sectional structures: (**A2**,**B2**); outer surface: (**A3**,**B3**); inner surface: (**A4**,**B4**); EDX elemental mapping images: (**C1**–**C4**).

Figure 4 presents the microscopic morphology of the green membrane and dense LWO-LSF dual-phase membrane. As illustrated in Figure 4(A1), the green membrane exhibits a well-defined four-channel structure. It displays an asymmetric shape, with distinctive features including sponge-like and finger-like pores (Figure 4(A2)), which are beneficial for reducing mass transfer resistances. A green membrane with outer and inner surfaces can be observed in Figure 4(A3,A4), both of which have a porous structure and particles distributed on the surface.

The microstructure of the dense LWO-LSF hollow fiber membrane is shown in Figure 4(B1–B4), which clearly exhibits a four-channel structure with outer and inner surfaces. In contrast with Figure 4(A2), the sponge-like structure is densified, and the finger-like pores contract, yet the structure remains intact in Figure 4(B2). Figure 4(B3,B4) further indicates that the inner and outer surfaces exhibit clear grain morphology and distinct grain boundaries. In addition, as depicted in Figure 4(C1–C4), EDX shows that the LWO and LSF phases are uniformly distributed, indicating good chemical compatibility between them. This result is consistent with the XRD analysis.

### 3.3. Thermal Sintering Test

**Figure 5:**
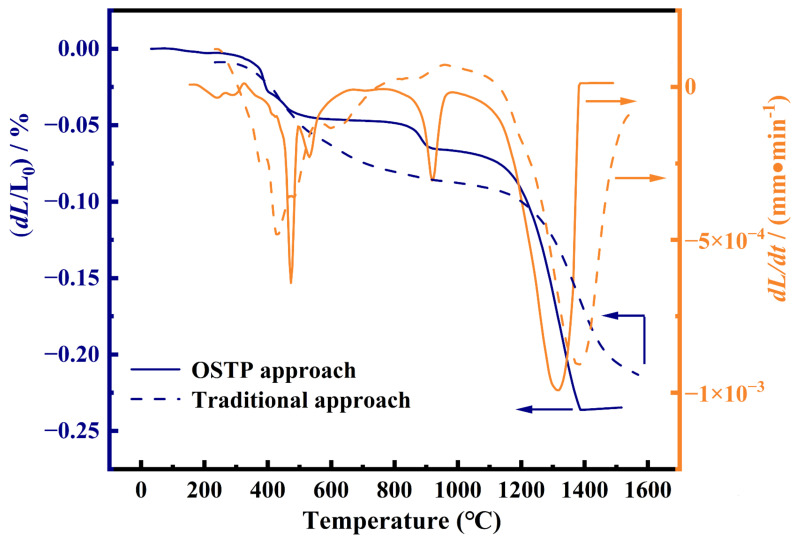
Temperature dependence of the sintering rate and corresponding differential curves for green LWO-LSF hollow fiber membranes fabricated via the one-step thermal treatment process (OSTP) and the traditional method.

Figure 5 presents the thermal sintering behavior of green LWO-LSF membranes fabricated via the one-step thermal treatment process (OSTP) and the conventional method. The preparation procedures of the one-step thermal processing (OSTP) and the conventional method have been detailed in our previous work [32]. As shown in Figure 5, the OSTP curve exhibits the characteristic features of each stage of the thermal sintering process over the temperature range from room temperature to 1500 °C. At 375 °C, the dual-phase membrane exhibits the first significant shrinkage, which corresponds to the evaporation of adsorbed water and the decomposition of polymers such as polyetherimide and polyvinylpyrrolidone [38]. This shrinkage ends at 525 °C, and the system enters the first sintering plateau. The second significant shrinkage indicates that the metal oxide components in the raw materials begin to undergo solid-phase reactions at 825 °C, with the fluorite and perovskite phases initially forming, marking the start of densification of the LWO-LSF dual-phase membrane [38,39,40]. After 1125 °C, the overall process enters a significant sintering stage, characterized by a sharp increase in the shrinkage rate, reflecting the close packing and rearrangement of the LWO and LSF particles. The sintering plateau occurs in the 1386–1500 °C range, illustrating that the system enters the complete densification stage. In comparison, traditional-prepared membranes exhibit mild shrinkage at 150–900 °C, with low shrinkage rates and minimal variation, driven by water evaporation, binder decomposition, and particle rearrangement—this is the pre-sintering stage without obvious grain growth or densification. From 1000 °C to 1250 °C, the slope of the shrinkage curve increases significantly, and densification continues until 1500 °C.

In terms of grain growth, the average grain size of OSTP-prepared membranes is considerably smaller than that of membranes from traditional multi-step sintering. In the traditional process [41], precursors are first sintered into separate phases, then shaped and sintered repeatedly at high temperatures, leading to severe grain coarsening and larger final grains. By contrast, OSTP realizes in situ reactive sintering, where phase formation and membrane sintering occur simultaneously, thus avoiding excessive grain growth from multiple high-temperature treatments.

### 3.4. LWO-LSF Hydrogen Permeation

**Figure 6:**
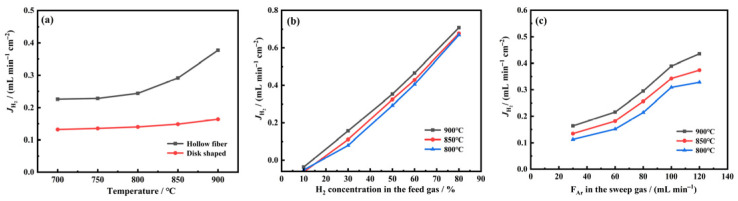
(**a**) Comparison of hydrogen fluxes between hollow fiber membranes and disk membranes. Operation condition: feed gas: 80 mL·min^−1^ of 50% H_2_/N_2_; sweep gas: 100 mL·min^−1^ of Ar. (**b**) LWO-LSF hollow fiber membrane with different feed H_2_ concentrations [100 mL·min^−1^ Ar sweep flow rate] and (**c**) LWO-LSF hollow fiber membrane with different Ar flow rates [80 mL·min^−1^ of 50% H_2_/N_2_]. Disk membrane thickness: 1 mm.

The effects of membrane microstructure, feed hydrogen concentration, and sweep gas flow rate on the hydrogen permeation flux of LWO-LSF hollow fiber membranes were investigated. Firstly, the effect of membrane configuration is discussed. The hydrogen partial pressure was calculated from the hydrogen pressure of the feed gas, which was maintained at 0.5 atm and kept constant throughout the permeation measurement. Figure 6a compares the hydrogen flux of the LWO-LSF hollow fiber and disk membrane under the same test conditions at 700–900 °C. For hollow fiber membranes, feed was introduced to the lumen side and sweep gas was applied to the shell side; for disk membranes, feed and sweep streams were arranged on opposite sides.

As depicted in Figure 6a, the hydrogen permeation fluxes of both hollow fiber and disk membranes exhibit a pronounced increase with elevating temperature, which is attributed to the thermally activated nature of both surface reactions and bulk ion diffusion, which are considerably accelerated at elevated temperatures [42]. At 900 °C, the flux attains 0.377 mL·min^−1^·cm^−2^ for the hollow fiber membrane and 0.163 mL·min^−1^·cm^−2^ for the disk membrane. The hollow fiber membrane exhibits a hydrogen flux approximately 2.3 times higher than that of the disk membrane. The higher hydrogen permeation flux of hollow fiber membranes is mainly ascribed to their considerably thinner effective separation layer (typically 100–200 μm). By contrast, the disk membrane used in this study has a thickness of 1 mm. The lower permeation resistance of hollow fiber membranes relative to disk membranes further enhances hydrogen permeation flux.

Subsequently, hydrogen partial pressure represents another key driving force for hydrogen permeation. Increasing the hydrogen concentration on the feed side or elevating the sweep gas flow rate to reduce the hydrogen partial pressure on the permeate side can both effectively enlarge the hydrogen partial pressure gradient across the membrane, thereby facilitating hydrogen permeation. At 900 °C with lumen-side feeding and shell-side sweeping for LWO-LSF hollow fiber membranes, Figure 6b shows that the H_2_ flux increases markedly from 0.156 to 0.708 mL·min^−1^·cm^−2^ as the feed hydrogen concentration is elevated from 30% to 80% (pH_2_ = 0.8 atm, 0.6 atm, 0.5 atm, 0.3 atm, and 0.1 atm, respectively). As depicted in Figure 6c, the H_2_ flux rises from 0.294 to 0.389 mL·min^−1^·cm^−2^ with increasing Ar sweep flow rate from 80 to 100 mL·min^−1^, the hydrogen partial pressure on the feed side is 0.5 atm and is kept constant throughout the experiments. These results demonstrate that hydrogen partial pressure significantly influences hydrogen permeation flux.

**Figure 7:**
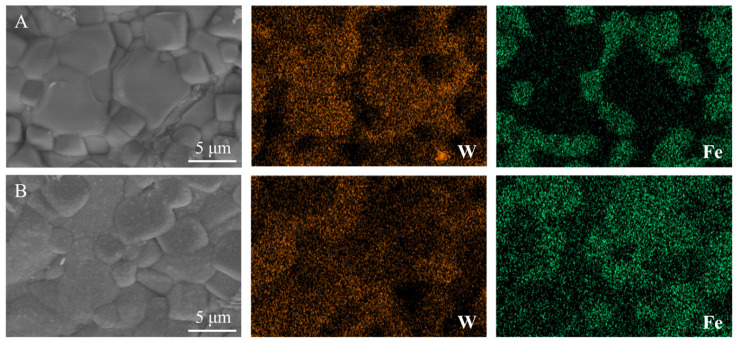
SEM and EDX images of the LWO-LSF hollow fiber membrane after hydrogen permeation: (**A**) sweep side; (**B**) feed side. Operation condition: 900 °C; feed gas: 80 mL·min^−1^ of 50% H_2_/N_2_ on lumen side; sweep gas: 100 mL·min^−1^ of Ar on shell side. Both sides are under a dry atmosphere.

Figure 7 displays the SEM and EDX images of the LWO-LSF hollow fiber after H_2_ permeation testing at high temperature. Here, A denotes the sweep side, and B denotes the feed side. From SEM micrographs, the structural integrity of the LWO-LSF membrane is well preserved on both the sweep and feed surfaces after H_2_ permeation, with a dense microstructure maintained on both sides. This confirms the excellent mechanical stability of the membrane under the experimental conditions of a 50% H_2_/N_2_ gas mixture.

As shown in the EDX elemental mapping, the feed side exhibits pronounced surface diffusion and migration of W and Fe under the strong reducing atmosphere, resulting in the blurring of elemental boundaries. The primary mechanism is that H_2_ modifies the membrane surface morphology and elemental distribution through reduction and diffusion, without compromising the membrane’s dense microstructure and chemical stability.

**Figure 8:**
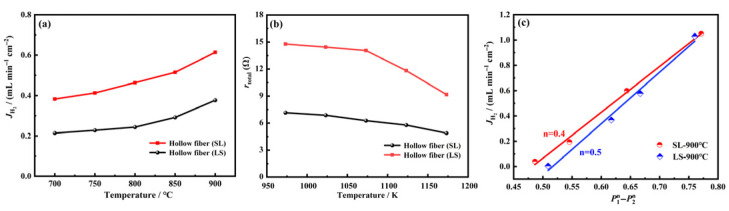
(**a**) Flux comparison between SL and LS for different hydrogen feed modes of LWO-LSF hollow fiber membrane. Operation condition: 900 °C; feed gas: 80 mL·min^−1^ of 50% H_2_/N_2_; sweep gas: 100 mL·min^−1^ of Ar. (**b**) Comparison of hydrogen permeation resistance of the LWO-LSF four-channel hollow fiber membrane under SL and LS modes at different temperatures. (**c**) Hydrogen flux of the LWO-LSF four-channel hollow fiber membrane as a function of $P_{1}^{n}-P_{2}^{n}$ under SL and LS modes (SL: P_1_ was set at 0.2, 0.3, 0.5, and 0.8 atm; LS: P_1_ was set at 0.3, 0.5, 0.6, and 0.8 atm).

Using a 50 vol% H_2_/N_2_ gas mixture as the feed gas, hydrogen permeation fluxes were measured under the two operating modes over the temperature range of 700–900 °C (Figure 8a). When the feed gas is introduced into the lumen side of the hollow fiber membrane and the sweep gas into the shell side, H_2_ permeates from the lumen to the shell; this configuration is designated as the LS mode. Conversely, in the SL mode, the sides for the sweep gas and H_2_ feed are reversed. These two modes represent distinct directions of H_2_ flow within the hollow fiber membrane. As shown in Figure 8a, the H_2_ permeation flux of the SL mode reaches 0.613 mL·min^−1^·cm^−2^ at 900 °C, while that of the LS mode is 0.377 mL·min^−1^·cm^−2^. The flux of the SL mode is 1.62 times that of the LS mode, indicating that the H_2_ permeation flux of the hollow fiber membrane in the SL mode is significantly higher than that in the LS mode.

To clearly explain the experimental results presented in Figure 8a, the permeation resistances of the LWO–LSF hollow fiber membrane under both SL and LS modes were calculated. Based on the hydrogen permeation resistance theory for mixed proton–electron conductors, the detailed formula is presented in Equation (2) [43], whose detailed procedure has been described in our previous work; the hydrogen mass transfer resistance was determined in the present study. As shown in Figure 8b, the hydrogen permeation resistance under both modes increases significantly with decreasing temperature, mainly because lower temperatures markedly suppress the rates of hydrogen bulk diffusion and surface exchange. The hydrogen permeation resistance in the SL mode is considerably lower than that in the LS mode. Since the flux is proportional to the inverse of the resistance and the driving force, the higher effective driving force in the SL mode explains the experimental results shown in Figure 8a.

(2) $$r_{\mathrm{total}}=\frac{\mathrm{RT}}{4F^{2}}\frac{1}{AJ_{H_{2}}}\ln\left(\frac{P_{1}}{P_{2}}\right)$$

To identify the rate-limiting step for hydrogen permeation through the LWO–LSF hollow fiber membrane under SL and LS modes, the dependence of hydrogen flux on driving force was examined at 900 °C. According to the rate-limiting theory for mixed proton–electron conductor (MPEC) membranes [44,45,46], Figure 8c shows that the partial pressure exponent *n* is 0.4 in the SL mode and 0.5 in the LS mode at 900 °C. This suggests that hydrogen permeation is co-controlled by surface exchange and bulk diffusion in the SL mode, but dominated by surface exchange reactions in the LS mode.

**Figure 9:**
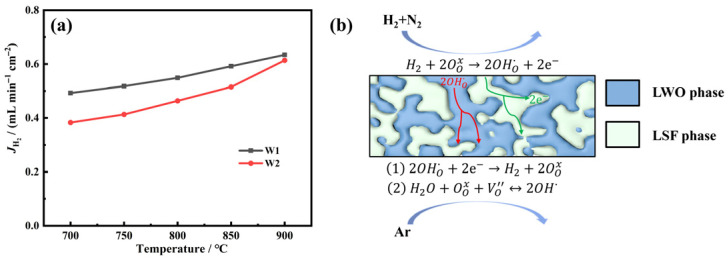
(**a**) Effect of 3% H_2_O addition on hydrogen flux in SL mode. W1: feed gas: 80 mL·min^−1^ of 50% H_2_/N_2_, sweep gas: 97 mL·min^−1^ of Ar and 3 mL·min^−1^ H_2_O; W2: 80 mL·min^−1^ of 50% H_2_/N_2_ as feed and 100 mL·min^−1^ of Ar as sweep. The feed side consists entirely of dry gases. (**b**) Schematic diagram of the hydrogen permeation mechanism under W1 conditions.

The effect of water vapor on the hydrogen permeation flux of the LWO-LSF hollow fiber membrane was investigated under the SL mode. As shown in Figure 9a, the hydrogen permeation flux under humidified sweep gas (W1) is higher than that under dry sweep gas (W2), reaching 0.634 mL·min^−1^·cm^−2^ at 900 °C. It should be clarified that introducing 3% H_2_O on the sweep side does not affect the hydrogen partial pressure, and the hydrogen partial pressure on the feed side is consistently maintained at 0.5 atm throughout all tests.

In the H_2_O-free atmosphere, the W2 system lacks additional proton carriers [35]. In contrast, under the W1 condition, water vapor on the sweep side decomposes into hydrogen and oxygen at elevated temperatures [47], resulting in higher hydrogen detection by gas chromatography. On the other hand, water vapor reacts with oxygen vacancies ($V_{O}^{\prime \prime }$) and lattice oxygen ($O_{O}^{x}$) in the membrane to generate more protonated lattice oxygen, thereby increasing the proton conductivity of the membrane material and further improving its hydrogen separation performance. The hydrogen permeation process with steam introduced on the sweep side is illustrated in Figure 9b. The hydrogen generated on the sweep side consists of two components, (1) and (2), where (1) originates from hydrogen on the feed side via transmembrane transport, and (2) is produced by the dissociation of water vapor on lattice oxygen at the sweep side.

**Figure 10:**
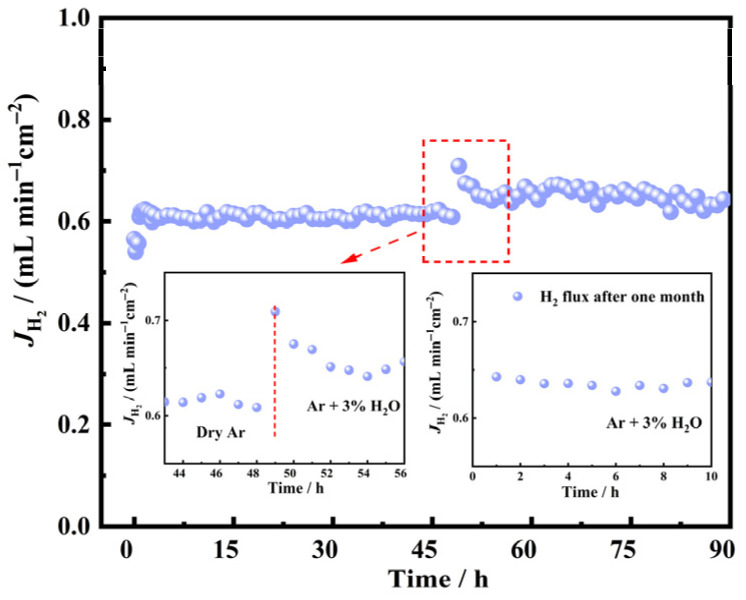
Long-time stability of the LWO-LSF hollow fiber membrane at 900 °C. 0–49 h: Both sides are under dry atmosphere; feed gas: 80 mL·min^−1^, containing 50% H_2_/N_2_; sweep gas: 100 mL·min^−1^ of Ar. 49–90 h: sweep gas: 97 mL·min^−1^ of Ar + 3 mL·min^−1^ H_2_O. 0–10 h: An additional 10 h stability test was conducted one month later under the same conditions as the 49–90 h long-term measurement.

The long-term operational stability of the dual-phase LWO-LSF four-channel hollow fiber membrane was evaluated to verify its potential for practical industrial applications. As illustrated in Figure 10, the membrane was operated in the SL mode at 900 °C under a dry atmosphere on both sides, delivering a stable hydrogen flux of 0.61 mL·min^−1^·cm^−2^ for 49 h. At the 49th hour, 3% H_2_O was introduced into the system, and the hydrogen flux remained essentially stable at approximately 0.63 mL·min^−1^·cm^−2^ throughout the subsequent 41 h of operation. After 1 month, a supplementary 10 h stability test was conducted on the membrane under identical operating conditions to those of the 49–90 h test. The results indicate that the hydrogen permeation flux remained stable at approximately 0.63 mL·min^−1^·cm^−2^, further verifying the excellent structural stability of the LWO-LSF hollow fiber membrane.

## 4. Conclusions

The dual-phase LWO-LSF four-channel hollow fiber membrane was prepared using a one-step heat-treatment method and phase transformation technology. In the LS mode, under the conditions of 900 °C and 50% H_2_/N_2_, the hydrogen permeation flux was 0.377 mL·min^−1^·cm^−2^, which was 2.3 times that of the disk-shaped membrane. In the SL mode, the hydrogen permeation flux increased to 0.613 mL·min^−1^·cm^−2^; when 3% H_2_O was added to the sweep side, the flux could reach 0.634 mL·min^−1^·cm^−2^. To further evaluate the stability of the LWO-LSF hollow fiber membrane, additional long-term stability testing was conducted with a feed gas of 50% H_2_/N_2_ and Ar on the sweep side. The LWO-LSF hollow fiber membrane maintained a hydrogen flux of 0.61 mL·min^−1^·cm^−2^ within 0 to 49 h at 900 °C in the SL mode. At the 49th hour, when 3% H_2_O was added on the sweep side, the hydrogen flux increased to 0.63 mL·min^−1^·cm^−2^, which remained stable until 90 h. One month later, the membrane operated continuously for 10 h under water-vapor-sweep conditions, and its hydrogen permeation flux remained stable at 0.63 mL·min^−1^·cm^−2^.

## Data Availability

The original contributions presented in this study are included in the article. Further inquiries can be directed to the corresponding author.
